# Systematic Characterization of Cancer‐Associated SPOP Mutants Reveals Novel and Reprogrammable Degradative Activities

**DOI:** 10.1002/cbic.202500914

**Published:** 2026-01-14

**Authors:** Alana G. Caldwell, Harshil Parmar, Xiaokang Jin, Chen Zhou, Xiaoyu Zhang

**Affiliations:** ^1^ Interdisciplinary Biological Sciences Graduate Program Northwestern University Evanston USA; ^2^ Department of Chemistry Northwestern University Evanston USA; ^3^ Chemistry of Life Processes Institute Northwestern University Evanston USA; ^4^ Robert H. Lurie Comprehensive Cancer Center Northwestern University Chicago USA; ^5^ Center for Human Immunobiology Northwestern University Chicago USA; ^6^ International Institute for Nanotechnology Northwestern University Evanston USA

**Keywords:** E3 ligase, protein–protein interactions, proteomics, recurrent mutation, targeted protein degradation

## Abstract

Speckle‐type POZ protein (SPOP) functions as the substrate adaptor of the Cullin3‐RING ligase complex and is recurrently mutated in multiple cancer types. Among these, F102C and F133L are frequent prostate cancer mutations within the substrate‐binding domain, yet their biochemical consequences remain incompletely understood. Using quantitative proteomics, we show that SPOP‐F133L, unlike SPOP‐F102C, retains degradative activity toward the nuclear basket proteins NUP153 and TPR, indicating substrate‐dependent loss‐of‐function. Moreover, SPOP‐F133L induces partial down‐regulation of p53 through a Cullin‐RING ligase‐dependent, post‐translational mechanism, revealing a potential neo‐substrate relationship. Finally, we demonstrate that both SPOP‐F102C and SPOP‐F133L support targeted protein degradation in an engineered cellular system. These findings define the degradative capacities of SPOP mutants and highlight opportunities to repurpose these variants as mutant‐selective E3 ligases for therapeutic applications.

## Graphical abstract

Quantitative proteomics reveal distinct activities of speckle‐type POZ protein (SPOP) cancer mutants. SPOP‐F133L, unlike F102C, retains degradation of NUP153 and TPR, and partially down‐regulates p53 through a Cullin‐RING ligase‐dependent mechanism. Both mutants support targeted protein degradation in engineered cells, highlighting opportunities to exploit SPOP variants for ligand‐induced protein degradation in cancers harboring these mutations.

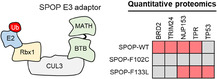

## 1 Introduction

The ubiquitin‐proteasome system is the primary pathway for the degradation of intracellular proteins, thereby maintaining proteome homeostasis [1]. Substrate specificity within this pathway is conferred by E3 ubiquitin ligases, which recruit target proteins to ubiquitination machinery [2]. Among these, the Cullin‐RING ligases (CRLs) represent the largest E3 family. They are modular assemblies comprised of 1) a Cullin scaffold, 2) a RING‐box protein such as Rbx1 that recruits E2 ubiquitin‐conjugating enzymes, and 3) a substrate‐recognition receptor [3]. Speckle‐type POZ protein (SPOP) serves as the substrate‐recognition component of the Cullin3‐RING ligase complex. The BTB domain of SPOP anchors it to the Cullin scaffold, whereas the MATH domain recognizes substrates, promoting their ubiquitination and degradation by the proteasome [4] (Figure S1A). SPOP localizes to nuclear speckles through higher‐order oligomerization, forming subnuclear hubs that promote efficient ubiquitination [5]. Functionally, SPOP is recognized as a tumor suppressor, as many of its established substrates are oncogenic regulators, such as BET family proteins (e.g., BRD2, BRD3, BRD4) [6], the androgen receptor [7], and the androgen signaling coactivator TRIM24 [8].

**FIGURE 1:**
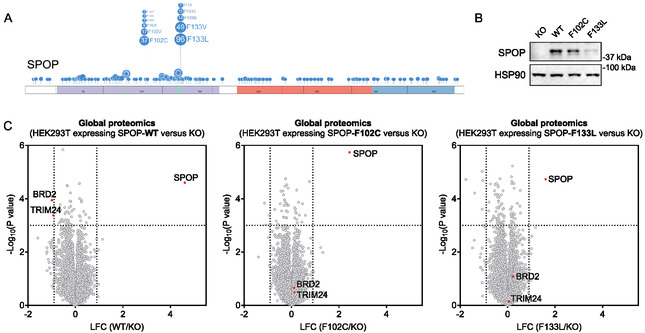
Generation of SPOP KO HEK293T cell models reconstituted with SPOP‐WT, F102C, or F133L. (A) Domain architecture of SPOP illustrating recurrent cancer‐associated mutations clustered within the substrate‐binding MATH domain. The F102C and F133L variants examined in this study are indicated. The schematic was generated using ProteinPaint (https://proteinpaint.stjude.org/). (B) Western blot analysis of SPOP expression in SPOP KO HEK293T cells and in KO cells stably expressing SPOP‐WT, F102C, or F133L. The result is representative of two experiments (*n* = 2 biologically independent samples). (C) Volcano plot showing global proteomic changes between SPOP KO HEK293T cells and KO cells stably expressing SPOP‐WT, F102C, or F133L (*n* = 4 biological independent samples). *P* values were calculated by two‐sided t‐test and adjusted using Benjamini–Hochberg correction for multiple comparisons.

Recurrent loss‐of‐function mutations in SPOP have been identified in multiple cancers, most prominently in prostate cancer, where such mutations occur in up to ∼15% of primary and metastatic cases [9, 10]. These mutations predominantly cluster within the substrate‐binding MATH domain, frequently occurring at residues Y87, F102, M117, W131, and F133 [11]. Among them, F102C and F133L are two of the most frequently observed SPOP mutations (Figure 1A). These mutations disrupt substrate binding, resulting in stabilization of oncogenic targets and promotion of tumorigenesis [11, 12]. Conversely, context‐dependent oncogenic functions of SPOP have been observed in other cancer types. For example, the SPOP‐M35L mutant identified in hepatocellular carcinoma increases affinity for the tumor suppressor IRF2BP2, leading to its enhanced ubiquitination and degradation, thereby promoting cell proliferation and metastasis [13]. Despite these insights, a comprehensive and proteome‐wide understanding of how recurrent SPOP mutations alter substrate engagement remains incomplete. In this study, we employ quantitative proteomics to systematically characterize the endogenous substrates and neo‐substrates perturbed by SPOP‐F102C and SPOP‐F133L. Moreover, we investigate whether recurrent SPOP mutations can be exploited as mutant‐selective E3 ligases for targeted protein degradation (TPD) applications.

## 2 Results and Discussion

### 2.1 Quantitative Proteomic Profiling of Protein Expression Changes Driven by SPOP‐Wild‐Type (WT), F102C, and F133L

Previous proteomic studies have primarily focused on identifying endogenous SPOP substrates and determining whether cancer‐associated SPOP mutants exhibit loss‐of‐function in degrading these substrates [14, 15, 16]. Given that SPOP mutations may both impair degradation of native substrates and promote degradation of neo‐substrates, we sought to systematically characterize these effects using mass spectrometry‐based whole‐proteome analysis. We first generated SPOP knockout (KO) HEK293T cells (Figure S1B). Consistent with the established role of SPOP in targeting BRD2 and TRIM24 for degradation [6, 8], both proteins were up‐regulated in SPOP KO cells, confirming functional disruption of SPOP in this cell model (Figure S1C and Table S1). We then reintroduced SPOP‐WT and two recurrent prostate cancer mutants, F102C and F133L, into the KO background to generate stable cell lines (Figure 1A,B). Proteomic profiling revealed that only SPOP‐WT, but not the F102C or F133L mutants, restored degradation of BRD2 and TRIM24 (Figure 1C and Table S2). Moreover, we observed a similar trend for BRD4, where SPOP‐WT, but not the F102C or F133L mutants, restored BRD4 degradation, while BRD3 levels remained unaffected (Figure S1D). These findings are consistent with previous studies indicating that both mutations are loss‐of‐function variants [16, 17, 18]. Thus, our reconstituted system provides a functional platform to further investigate substrate specificity and potential neo‐substrate recognition by SPOP mutants.

### 2.2 SPOP‐F133L Retains Degradative Activity Toward Nuclear Basket Proteins NUP153 and TPR

**FIGURE 2:**
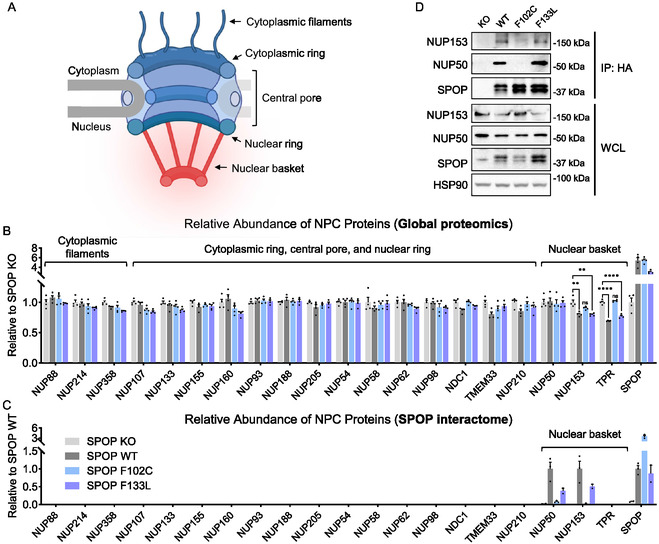
SPOP‐F133L, but not SPOP‐F102C, retains degradative activity toward nuclear basket proteins NUP153 and TPR. (A) Schematic illustration of the nuclear pore complex showing the cytoplasmic, central, and nuclear basket regions. The schematic was created with BioRender.com. (B) Quantitative proteomic analysis of 20 nucleoporins in SPOP KO HEK293T cells reconstituted with SPOP‐WT, F102C, or F133L. Data are presented as mean ± SEM (*n* = 4 biological independent samples). Statistical significance was assessed using unpaired two‐tailed Student's t‐tests. The color legend for each bar is shown in Figure 2C. (C) AP‐MS analysis of SPOP‐WT, F102C, and F133L. Data are presented as mean ± SEM (*n* = 2 biologically independent samples for SPOP KO and F133L, *n* = 3 biologically independent samples for SPOP‐WT and F102C). (D) Co‐immunoprecipitation analysis showing the interaction between HA‐tagged SPOP and NUP153 or NUP50. The result is representative of two independent experiments (*n* = 2 biologically independent samples).

The human nuclear pore complex (NPC) is a large macromolecular assembly composed of a number of distinct proteins known as nucleoporins [19] (Figure 2A). A previous study identified NUP153 as a substrate of SPOP, suggesting a role for SPOP in regulating NPC function and potentially protein transport through the nuclear envelope [20]. The study further showed that the SPOP‐F102C mutant loses the ability to degrade NUP153. Using our established cell models, we sought to investigate whether SPOP targets additional components of the NPC and whether SPOP‐F133L similarly acts as a loss‐of‐function mutant unable to degrade nucleoporins.

Our whole‐proteome analysis quantified 20 nucleoporins, containing all major NPC substructures, including the cytoplasmic filaments, cytoplasmic ring, central pore, nuclear ring, and nuclear basket (Figure 2B and Table S2). Among these, the nuclear basket protein NUP153, which is a known SPOP substrate, was reduced upon SPOP‐WT expression (Figure 2B). Notably, the abundance of another nuclear basket component, TPR, was also reduced under the same conditions (Figure 2B), indicating that TPR is a potential new substrate of SPOP. In fact, NUP153 and TPR were the only nucleoporins among all quantified proteins that were down‐regulated in cells expressing SPOP‐WT. This suggests that SPOP may specifically regulate components of the nuclear basket within the NPC. Consistent with previous findings, the SPOP‐F102C mutant lost the ability to degrade these nuclear basket proteins (Figure 2B and Table S2). Surprisingly, SPOP‐F133L retained degradative activity toward both NUP153 and TPR (Figure 2B and Table S2), indicating that it may be a substrate‐dependent loss‐of‐function mutant. These results also suggest that in cancer cells harboring the SPOP‐F133L mutation, the expression levels of NUP153 and TPR may remain largely unaffected, potentially preserving nuclear basket function.

To further investigate the mechanism underlying the loss of degradative activity in SPOP‐F102C compared with SPOP‐F133L, we performed affinity purification‐mass spectrometry (AP‐MS) using SPOP as the bait (Figure S2 and Table S3). The results showed that both SPOP‐WT and SPOP‐F133L interacted with NUP153, whereas SPOP‐F102C did not (Figure 2C, S2), suggesting that the impaired degradation of NUP153 by SPOP‐F102C may result from the loss of physical interaction. This difference likely reflects the distinct biochemical consequences of the two amino‐acid substitutions, namely that substitution of phenylalanine with cysteine at position 102 introduces a smaller and more polar side chain that disrupts the local hydrophobic packing critical for substrate recognition, whereas substitution with leucine at position 133 may largely preserves hydrophobicity and side‐chain volume, causing only minimal perturbation of the NUP153‐binding interface. These findings highlight an interesting avenue for future structural studies on the molecular basis of SPOP‐substrate interactions. Our AP‐MS did not detect TPR, possibly due to weak or transient binding between SPOP and TPR. Future work employing approaches optimized to capture such transient interactions could provide further insight. In addition, the AP‐MS identified NUP50 as another interactor of SPOP‐WT and SPOP‐F133L, but not SPOP‐F102C (Figure 2C, S2). This interaction did not lead to substrate degradation in the whole‐proteome analysis (Figure 2B), consistent with prior reports that SPOP binds but does not degrade NUP50 [20]. Moreover, these interactions identified by AP‐MS were validated by co‐immunoprecipitation using HA‐tagged SPOP constructs (Figure 2D).

Computational modeling, including AlphaFold‐based predictions, could in principle provide insight into how SPOP mutations alter substrate engagement. However, while the SPOP MATH domain is well resolved and confidently predicted by AlphaFold, the substrates examined here (BRD2, BRD4, TRIM24, NUP153, and TPR) contain extensive intrinsically disordered or low‐confidence regions. These features make reliable complex modeling challenging and limit the interpretability of the predicted interfaces. Future structural studies and advances in modeling tools will be important for more accurately elucidating how SPOP mutations differentially affect substrate recognition.

### 2.3 p53 Is Down‐Regulated in SPOP‐F133L‐Expressing Cells Through a CRL‐Dependent Post‐Translational Mechanism

Despite being generally considered loss‐of‐function mutations, our data suggest that SPOP‐F133L may retain degradative activity toward certain substrates. We also considered the possibility that these mutants might acquire gain‐of‐function properties by recognizing and degrading neo‐substrates. To investigate this, we sought to identify proteins that are specifically down‐regulated in cells expressing SPOP mutants but not in those expressing SPOP‐WT.

**FIGURE 3:**
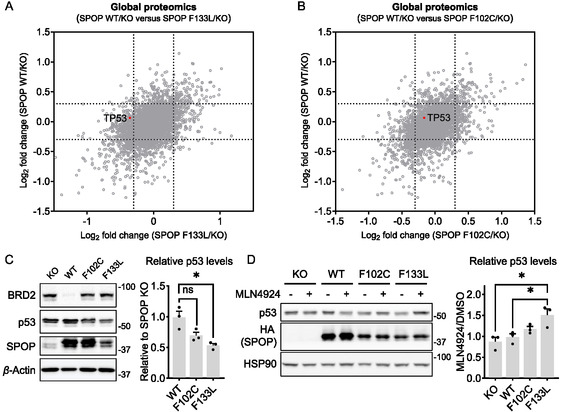
p53 is down‐regulated in SPOP‐F133L‐expressing cells through a CRL‐dependent post‐translational mechanism. (A) Global proteomic analysis comparing two calculated ratios, SPOP‐WT/KO and SPOP‐F133L/KO, to identify proteins differentially affected by the SPOP‐F133L mutant. (B) Global proteomic analysis comparing two calculated ratios, SPOP‐WT/KO and SPOP‐F102C/KO, to identify proteins differentially affected by the SPOP‐F102C mutant. (C) Western blot analysis of p53 expression in SPOP KO HEK293T cells and in KO cells stably expressing SPOP‐WT, F102C, or F133L. BRD2 serves as a positive control. The result is representative of three experiments. The bar graph represents quantification of p53 protein levels normalized to *β*‐Actin. Data are presented as mean ± SEM (*n* = 3 biological independent samples). Statistical significance was assessed using unpaired two‐tailed Student's t‐tests (*P* = 0.014 for WT versus F133L). (D) Western blot analysis of p53 expression in SPOP KO HEK293T cells and in KO cells stably expressing SPOP‐WT, F102C, or F133, with or without treatment with 2 μM MLN4924 for 4 h. The result is representative of three experiments. The bar graph shows the quantification of p53 protein levels (normalized to *β*‐Actin) in MLN4924‐treated versus DMSO‐treated cells. Data are presented as mean ± SEM (*n* = 3 biological independent samples). Statistical significance was assessed using unpaired two‐tailed Student's t‐tests (*P* = 0.025 for KO versus F133L; *P* = 0.036 for WT versus F133L).

Because SPOP regulates a number of transcriptional factors and coregulators [21], resulting in broad proteomic alterations, changes observed in whole‐proteome analyses could arise from either transcriptional or post‐translational effects. Therefore, we focused on functionally important proteins that showed differential expressions and aimed to validate whether these changes occur through post‐translational regulation. Among these candidates, p53 caught our attention as a well‐characterized tumor suppressor and transcription factor that regulates cell‐cycle arrest, DNA repair, apoptosis, and senescence in response to cellular stress [22]. Notably, SPOP‐WT and SPOP‐F102C did not alter p53 protein levels, whereas SPOP‐F133L expression led to modest down‐regulation of p53 (Figure 3A,B). This observation was further confirmed by Western blot analysis (Figure 3C). Given that SPOP functions as part of a CRL E3 complex requiring Cullin neddylation for activity, we employed MLN4924, a selective inhibitor that blocks CRL activation by preventing Cullin neddylation [23], to assess whether the down‐regulation of p53 depends on CRL activity. Treatment with MLN4924 rescued the p53 down‐regulation induced by SPOP‐F133L (Figure 3D), indicating that this effect occurs via a post‐translational mechanism mediated by a CRL E3 ligase.

p53 is a master regulator of various pathways that suppress tumorigenesis [22]. To determine whether modest down‐regulation of p53 by SPOP‐F133L leads to measurable phenotypic changes, we assessed several p53‐dependent cellular processes, including cell proliferation, sensitivity to chemotherapeutic agents, and DNA damage‐induced cell‐cycle arrest, in cells expressing SPOP‐WT, SPOP‐F102C, or SPOP‐F133L. We first monitored cell proliferation over 96 h and observed no significant differences in growth rate among the four cell models (Figure S3A). We next tested cellular responses to doxorubicin, a chemotherapeutic agent that induces DNA double‐strand breaks and oxidative stress, thereby activating the p53 pathway and triggering cell‐cycle arrest and apoptosis [24]. In principle, cells with reduced p53 levels may show increased resistance to doxorubicin‐induced apoptosis. However, cytotoxicity assays revealed no significant shift in IC_50_ values between SPOP‐WT and SPOP‐F133L‐expressing cells (Figure S3B). Similarly, cell‐cycle arrest assays following doxorubicin treatment showed no significant differences among these cells (Figure S3C,D). We speculate that two explanations may account for the lack of detectable phenotypic changes following p53 down‐regulation. First, the extent of down‐regulation was relatively mild, with p53 protein levels reduced by 22% in SPOP‐F133L‐expressing cells compared with SPOP KO cells (*P* value = 0.005), which may be insufficient to elicit strong alterations in p53‐related pathways. Second, HEK293T cells may not represent an optimal system for evaluating tumorigenic phenotypes. Nonetheless, our findings highlight a potentially new regulatory mechanism in SPOP‐F133L‐expressing cells, thereby providing a basis for future investigation of this pathway in additional cancer‐relevant models.

### 2.4 SPOP‐F102C and SPOP‐F133L are TPD‐Competent E3 Ligases

**FIGURE 4:**
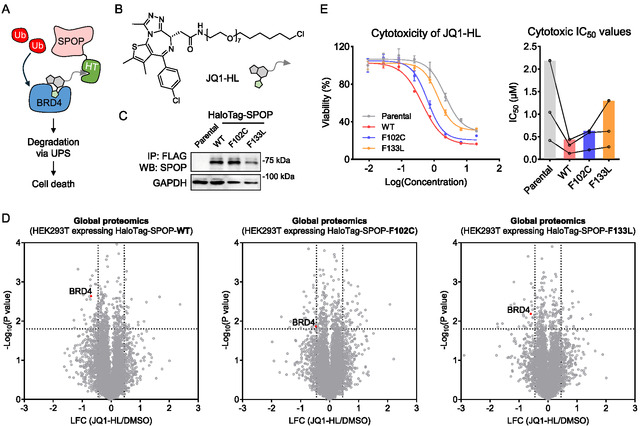
SPOP‐F102C and SPOP‐F133L support targeted protein degradation. (A) Schematic illustration of SPOP‐mediated BRD4 degradation, in which HaloTag (HT)‐fused SPOP and a bifunctional degrader cooperatively recruit BRD4 for proteasomal degradation. (B) Structure of JQ1‐HL. (C) Western blot analysis of SPOP expression in parental HEK293T cells and in HEK293T cells stably expressing HaloTag‐SPOP‐WT, F102C, or F133L. The result is representative of two experiments (*n* = 2 biologically independent samples). (D) Volcano plot showing global proteomic changes in HEK293T cells stably expressing HaloTag‐SPOP‐WT, F102C, or F133L, with or without JQ1‐HL treatment (1 µM, 24 h). Protein expression in HaloTag‐SPOP‐expressing cells treated with JQ1‐HL was normalized to the BRD4 ratio in HEK293T parental cells treated with JQ1‐HL versus DMSO. *P* values were calculated by two‐sided t‐test and adjusted using Benjamini–Hochberg correction for multiple comparisons (*n* = 2 biological independent samples). (E) Dose‐response curves and IC_50_ values of JQ1‐HL‐induced cytotoxicity in parental HEK293T cells and HEK293T cells stably expressing HaloTag‐SPOP‐WT, F102C, or F133L. Data are presented as mean ± SEM (*n* = 3 biological independent samples).

TPD has emerged as a powerful approach to manipulate protein homeostasis. By using small molecules or biologics to link target proteins to endogenous degradation systems such as the proteasome or lysosome, TPD enables their selective and efficient removal [25, 26]. SPOP is one of the several TPD‐competent E3 ligases and has been demonstrated to support biologics‐mediated protein degradation [27, 28, 29, 30, 31]. We recently introduced the concept of exploiting the somatic mutant FBXW7‐R465C for TPD, highlighting the potential to develop cancer‐selective degraders [32]. Building on this idea, we asked whether SPOP mutants might similarly serve as TPD‐competent ligases. As a proof‐of‐concept, we generated HaloTag‐fused SPOP‐WT and mutant constructs (Figure 4A) and synthesized JQ1‐HL, a bifunctional compound that links a HaloTag‐reactive chloroalkane ligand [33] to the BRD4 inhibitor JQ1 [34] (Figure 4B). This system allows us to assess BRD4 degradation as well as the resulting cytotoxicity, given that BRD4 is an essential gene whose loss can induce cell death [35].

We established HEK293T cell lines stably expressing HaloTag‐SPOP‐WT, F102C, or F133L (Figure 4C) and performed whole‐proteome analysis following treatment with JQ1‐HL. In all three models, JQ1‐HL treatment induced significant BRD4 degradation (Figure 4D and Table S4). Furthermore, this degradation translated into enhanced cytotoxicity, as reflected by decreased IC_50_ values for JQ1‐HL in cells expressing HaloTag‐SPOP (Figure 4E). Together, these findings suggest that SPOP‐F102C and SPOP‐F133L are capable of supporting TPD. Future studies exploring both heterobifunctional and molecular glue degraders may yield new ligands that hijack these SPOP mutants to drive selective target degradation in tumors harboring these mutations.

## 3 Conclusion

In this study, we combined systematic proteomic and functional analyses to reveal that SPOP‐F133L, but not SPOP‐F102C, retains degradative activity toward the nuclear basket proteins NUP153 and TPR, and can partially reduce p53 levels through a CRL‐dependent post‐translational mechanism. In addition, both mutants were able to support TPD in an engineered cellular system, suggesting that they may retain functional competence in mediating ligand‐induced degradation despite altered substrate recognition. Together, these findings expand our understanding of SPOP mutation‐associated functional diversity and suggest that certain cancer‐associated variants might be repurposed as TPD‐competent E3 ligases for therapeutic applications.

## Supporting Information

Additional supporting information can be found online in the Supporting Information section. The authors have cited additional references within the Supporting Information. [37]. **Supporting Fig. S1:** Generation and characterization of SPOP KO HEK293T cells. A. Schematic representation of the SPOP‐CUL3‐Rbx1 E3 ligase complex. B. Western blot analysis of SPOP expression in parental and SPOP KO HEK293T cells. The result is representative of two experiments (*n* = 2 biologically independent samples). C. Volcano plot showing global proteomic changes between parental and SPOP KO HEK293T cells (*n* = 4 biological independent samples for parental cells, *n* = 3 biological independent samples for SPOP KO cells). *P* values were calculated by two‐sided t‐test and adjusted using Benjamini‐Hochberg correction for multiple comparisons. LFC, log2 fold change. D. Bar graph showing TMT intensities of BRD2, BRD3, and BRD4 in SPOP KO HEK293T cells and KO cells stably expressing SPOP‐WT, F102C, or F133L (*n* = 4 biologically independent samples). *P* values were calculated by two‐sided t‐test. **Supporting Fig. S2:** Volcano plot showing the SPOP interactome compared with mock‐transfected cells (*n* = 2 biologically independent samples for mock and SPOP‐F133L, *n* = 3 biologically independent samples for SPOP‐WT and F102C). *P* values were determined using a two‐sided t‐test and adjusted for multiple comparisons using the Benjamini Hochberg method. **Supporting Fig. S3:** Partial down‐regulation of p53 by SPOP‐F133L does not produce detectable phenotypic changes. A. Cell proliferation curves of SPOP KO HEK293T cells and KO cells stably expressing SPOP‐WT, F102C, or F133L, measured over 96 hours by cell viability assays. Data are presented as mean ± SEM (*n* = 3). B. Dose response curves showing cell viability following treatment with Doxorubicin. IC50 values were calculated using nonlinear regression analysis. Data are presented as mean ± SEM (*n* = 3). C. Representative flow cytometry profiles of cell‐cycle distribution following treatment with Doxorubicin (1 μM, 24 hours). D. Quantification of cell‐cycle phases (G1, S, and G2/M) from (C) showing no significant differences among the four cell models. **Supporting Table S1:** Global proteomics comparing protein expression in HEK293T parental and SPOP KO cells. **Supporting Table S2:** Global proteomics comparing protein expression in SPOP KO HEK293T cells and KO cells stably expressing SPOP‐WT, F102C, or F133L. **Supporting Table S3:** AP‐MS analysis of the SPOP interactome in HEK293T cells expressing HA‐tagged SPOP‐WT, F102C, or F133L. **Supporting** **Table S4:** Global proteomics comparing protein expression in HEK293T cells stably expressing HaloTag‐SPOP‐WT, F102C, or F133L, with or without JQ1‐HL treatment.

## Funding

This study was supported by National Institute of General Medical Sciences (R35GM154945), Dr. Ralph and Marian Falk Medical Research Trust, Feinberg School of Medicine.

## Conflicts of Interest

The authors declare no conflicts of interest.

## Supporting information

Supplementary Material

## Data Availability

The mass spectrometry proteomics data have been deposited to the ProteomeXchange Consortium via the PRIDE [36] partner repository with the dataset identifier PXD071054.
